# Supplementary material for: Educational interventions to improve pediatric emergency care: A qualitative assessment of the perspectives of African healthcare workers
Source: PLOS Glob Public Health. 2025 Jan 7;5(1):e0004095. doi: 10.1371/journal.pgph.0004095 (PMC11706391; doi:10.1371/journal.pgph.0004095)
Supplement: S1 Appendix — (DOCX) [file pgph.0004095.s001.docx]

S1 Appendix. Recruitment Email.

 EMAIL:   *Seeking collaborators for mixed methods/ qualitative assessment of the AFEM Paediatric curriculum*

Dear AFEM Members,

Some members of the pediatric curriculum writing group for AFEM have been working on developing curricula for training in the management of pediatric emergencies that can be used in AFEM member countries for doctors, nurses, and mid-level providers who take care of sick children.  Short courses have been piloted in two different formats in Tanzania and Liberia.  We want to extend the training further, but first want to gain the perspective from sites where the training may occur on what they would find most useful, how they want to do the training, ways we can assure sustainability, and potential unintended consequences of the training we should try and avoid.  We are planning to develop a survey tool as well as an interview and/or focus group tool to obtain this information.  We are designing this using a mixed methods/qualitative approach and will submit for IRB approval before gathering data.  We would love to include author collaborators from the AFEM research and/or education committees in this project! If you would be interested in collaborating on this work, please contact Emily and Megan at [INSERT EMAIL ADDRESSES].

Sincerely,

Emily Hartford and Megan Schultz
