# Supplementary material for: Educational interventions to improve pediatric emergency care: A qualitative assessment of the perspectives of African healthcare workers
Source: PLOS Glob Public Health. 2025 Jan 7;5(1):e0004095. doi: 10.1371/journal.pgph.0004095 (PMC11706391; doi:10.1371/journal.pgph.0004095)
Supplement: S2 Appendix — (DOCX) [file pgph.0004095.s002.docx]

S2 Appendix. Interview Tool

Pediatric Emergency Medicine Training in Africa

Semi-Structured Interview Questions 

**I. Introduction**

Thank you for agreeing to participate in this interview about pediatric emergency care training.

This interview is being conducted as part of a study that aims to understand how best to provide training for health care providers in pediatric emergency care in African countries. To this end we are interviewing front-line providers, like you, who care for and teach others about caring for critically ill and injured children.  We hope to represent a variety of settings and countries across the African continent. Your participation is voluntary.  Your answers will be kept anonymous. At any point during the interview, you can decide that you want to stop participating. We will provide our contact information in case you have any concerns or questions in the future.

Do you consent to participate?

We’ll start by introducing ourselves and providing you some information about the interview.

**a) Introductions**

Introduce yourself as the interviewer

- Your name
- Your role and institution/location

Ask interviewee to introduce themselves:

- First name
- Healthcare profession (e.g. nurse, physician)
- Practice setting (hospital or clinic type)

**b) We will now provide you some information about the interview so you know what to expect:**

- **Length**: The interview is expected to last approximately 1 hour.
- **Recording**: We will be recording our discussion to have it later transcribed to be qualitatively analyzed.
- **Anonymity**: The data collected will be de-identified prior to analysis. Nothing will be directly attributed to you without seeking your permission directly beforehand.
- **Clarity**: Please feel free to interrupt at any time if terms used or questions asked are unclear.
- **Consent**: Your participation is voluntary and you can decide not to participate at any time.  Do we have your consent to participate in and record this interview?
- Do you have any questions before we start?

**II. Main Interview**

We will start by asking some questions about your background and practice.

**a) Background**

1. Where did you complete your (medical/nursing) training?
2. Where do you work now?
3. Country
4. Healthcare facility
5. Level of care (health center/district hospital/secondary/tertiary)
6. Description of facility (Public/Private/NGO, mission or charity/University hospital)
7. How many years have you worked in patient care?

**b) Clinical and Training Questions**

Now I will ask questions about training for pediatric emergencies (for example: respiratory distress, trauma, shock, seizure)

1. How often do you care for pediatric emergencies?

1. How prepared do you feel taking care of pediatric emergency patients?

*Prompt if needed:* Why do you not feel as prepared?

1. What has helped you to feel prepared with taking care of these patients?

1. Do you think additional training in pediatric emergencies would be beneficial?  Why or why not?

1. Have you ever taught a course about pediatric emergencies?
2. If so, which one(s)?
3. If so, how often?
4. If so, to whom?

Questions about format:

1. I’m going to read you a list of different ways to learn. Let me know if you’ve participated in any of them, and what you like or do not like about any of them. *(pros/cons)*

- Didactic lectures
- Interactive training (such as case discussions, group work, etc.)
- Simulation cases
- Short, in-person focused trainings less than a week long
- Weekly, ongoing/longitudinal training
- Remote learning
- Live lectures online
- Recorded lectures
- Workshop (interactive) online
- Self-guided online course, case-based program or app

What makes for the best type of course and why?

1. What do you think is the ideal format for a training? *(Example prompts: lectures or simulation; full days vs half-days; intensive short course vs intermittent longitudinal course)*

Questions about teachers:

1. A “train the trainer” model is when one person is trained as an expert in a topic and then goes on to train his or her colleagues.  Do you think the “train the trainer” model would work well to teach about pediatric emergencies in your setting?  Why or why not?

1. Have you ever received training or taught a course using the “train the trainer” model?

1. Is there someone already at your facility who teaches about pediatric emergencies?
2. If so, who?
3. If not, would you or someone you know, be interested and able to teach about pediatric emergencies?
4. Would it be helpful if teaching material (such as lecture slides or discussion cases) were provided to an instructor?

1. What do you like and not like about visiting trainers (for example, trainers from the USA) teaching pediatric emergencies in your setting?

1. Would you prefer to have a local or visiting provider teach in your setting? Why?

Questions about knowledge retention:

1. After learning about pediatric emergencies, what do you think helps people remember what they learned?

Questions about adverse consequences:

1. Do you think patient care is affected while you or your colleagues are away at a training?  Why or why not?
2. If yes, how is patient care affected?

1. Do visiting trainers or curricula developed in other countries ever recommend supplies and equipment that are not routinely available at your site?
2. If so, how do you handle that?

1. Do visiting trainers teach anything that conflicts with your local protocols or previous training?
2. If so, can you tell me about such an experience?

1. Any other problems about training courses that you’d like to share?

1. How would you try to prevent these negative consequences?

**Summary:**

1. If you could make your own course about pediatric emergences, what would it look like?

1. Please describe the resources you would need to implement this curriculum.

1. Please describe the barriers that would prevent you from being able to implement this curriculum.

**Conclusion**

Thank you for taking the time to share your experiences.  Is there anything else you want to tell me that I didn’t ask about?

- Will I be informed about the results?
- When the study is complete and published, you can receive a copy of the article by emailing any of us. All results will also be used to shape future AFEM Paeds curricula.
- How will I receive my compensation?
- $25 online gift card

Thank you for your time and contributions to this work.  If you have any questions or think of additional information, please contact Megan Schultz at [mlschultz@mcw.edu](mailto:mlschultz@mcw.edu) anytime. Best wishes for your ongoing work.
