# Supplementary material for: Educational interventions to improve pediatric emergency care: A qualitative assessment of the perspectives of African healthcare workers
Source: PLOS Glob Public Health. 2025 Jan 7;5(1):e0004095. doi: 10.1371/journal.pgph.0004095 (PMC11706391; doi:10.1371/journal.pgph.0004095)
Supplement: Attachment — Submitted filename: PLOS GH response to reviewers.docx [file pgph.0004095.s004.docx]

*Educational interventions to improve pediatric emergency care: a qualitative assessment of the perspectives of African healthcare workers*

Editor and reviewer comments and responses

August 7, 2024

Journal Requirements:
1.  Please include a complete copy of PLOS’ questionnaire on inclusivity in global research in your revised manuscript. Our policy for research in this area aims to improve transparency in the reporting of research performed outside of researchers’ own country or community. The policy applies to researchers who have travelled to a different country to conduct research, research with Indigenous populations or their lands, and research on cultural artefacts. The questionnaire can also be requested at the journal’s discretion for any other submissions, even if these conditions are not met.  Please find more information on the policy and a link to download a blank copy of the questionnaire here: [https://journals.plos.org/globalpublichealth/s/best-practices-in-research-reporting](https://urldefense.com/v3/__https:/journals.plos.org/globalpublichealth/s/best-practices-in-research-reporting__;!!NuzbfyPwt6ZyPHQ!vebrX6Da0irElz3W_ZuuegNOFgQD1HCGZTEl7sfVgUgNPX0voc4y2bTxPhXQF9vSCCPZ2RLu2rsk3vfXqbBfDk2T1KPP$). Please upload a completed version of your questionnaire as Supporting Information when you resubmit your manuscript.

We have completed this form and uploaded it under supporting information.

2. We do not publish any copyright or trademark symbols that usually accompany proprietary names, eg (R), (C), or TM  (e.g. next to drug or reagent names). Please remove all instances of trademark/copyright symbols throughout the text, including ® on page 17.

Thank you for noticing this, we have removed the symbol in this version.

3. "In the online submission form, you indicated that Data can by accessed by request via a link to de-identified interview transcripts.

 All PLOS journals now require all data underlying the findings described in their manuscript to be freely available to other researchers, either 
a. In a public repository, 
b. Within the manuscript itself, or 
c. Uploaded as supplementary information.

This policy applies to all data except where public deposition would breach compliance with the protocol approved by your research ethics board. If your data cannot be made publicly available for ethical or legal reasons (e.g., public availability would compromise patient privacy), please explain your reasons by return email and your exemption request will be escalated to the editor for approval. Your exemption request will be handled independently and will not hold up the peer review process, but will need to be resolved should your manuscript be accepted for publication. One of the Editorial team will then be in touch if there are any issues."

We have requested an exemption via email from uploading our data to preserve the anonymity of our participants. We look forward to your response.

Reviewer Comments to the Author

Reviewer #1: Thank you for the opportunity to review this article. I find it to be a very interesting and well-conducted qualitative research study. The findings are useful and reproducible in other low-resource countries. Although teaching through residencies is the ideal method, the insufficient number of these programs is a reality in many low- and middle-income countries. This adds interest to the research presented by the authors.

I believe the research successfully evaluates the perception of the short courses received and the reality in which they are implemented, which adds even greater value to the information obtained.
The reduction of staff during training sessions when the training frequency is not sustained and scheduled throughout the year, allowing protected time for training without affecting patient safety, is a very interesting point.

Although the authors consider the selection of respondents and their findings to be a limitation due to the sample obtained, I believe these are logical aspects that can be generalized to other low-income countries and continents. On the other hand, the selection of respondents likely gathered the opinions of providers involved and interested in learning and improving their skills; rather than being a limitation, these are probably individuals who have more tools to evaluate the items presented in the survey.

For all the above reasons, I consider that the research should be published.

My proficiency in English as a second language does not allow me to make stylistic corrections. I do not understand the following in line 139: author ES?

Thank you very much for your review of our manuscript, we appreciate your thoughts and considerations. Author “ES” is an abbreviation for the author Elena Seifert who completed the uploading of interview transcripts.

Reviewer #2: REVIEWER’S REPORT

TITLE: EDUCATIONAL INTERVENTIONS TO IMPROVE PEDIATRIC EMERGENCY CARE: A QUALITATIVE
ASSESSMENT OF THE PERSPECTIVES OF AFRICAN HEALTHCARE WORKERS

Thank you for giving me the opportunity to review this paper.

I commend the authors for contributing to the body of knowledge concerning educational interventions to improve paediatric emergency care in Africa. I reviewed the manuscript using the Consolidated Criteria for Reporting Qualitative Research (COREQ) guidelines: Tong A, Sainsbury P, Craig J. Consolidated criteria for reporting qualitative research (COREQ): a 32-item checklist for interviews and focus groups. International journal for quality in health care. 2007 Dec 1;19(6):349-57.

Thank you very much for your review.

TITLE
Appropriate

ABSTRACT
The authors should include the study limitations in the abstract.

INTRODUCTION
The introduction provides enough background information for the study, and addresses why it should be done.

METHODOLOGY

Interviewer and Participant Recruitment
How were the key informants selected? Where there any challenges during the selection process or any reasons given for non-participation?
The relationship and extent of interaction between the four selected interviewers and their key informants should be described.
In addition, the personal characteristics of the interviewers and their assumptions or personal interests in the research topic, if any, should be stated.

Thank you for these comments, we have updated the manuscript to provide additional details about the interviewers and on how key informants were selected. The interviewers did not have difficulty in recruiting colleagues to interview. There were no specific reasons given for non-participation. We added more of a description of the relationship between interviewers and key informants. Please see lines 126-129.

Study Procedures
Please provide information about duration of the interviews.

Interviews were approximately one hour, we have added this in the text in line 131.

Analyses
The use of multiple coders is commendable. Did the authors obtain feedback from key informants on the research findings?

We did not have the opportunity to get feedback from the key informants themselves as we did not obtain their contact information for the study.

RESULTS
Clearly presented. However, for supporting quotations, you may wish to include quotations from different participants.

We appreciate this suggestion. We felt the results tables were already quite long given the number of themes we report, and it might be difficult to include additional illustrative quotes. The quotes included do represent a variety of different key informants and not just one respondent.

DISCUSSION
Adequate.

General comments
This is an important study concerning educational interventions to improve pediatric emergency care within the region.
Please avoid beginning sentences with abbreviations. The authors may also wish to consider an English proofreading service.

Thank you very much for your detailed review! We have corrected any sentences that begin with abbreviations. Additionally, we have proofread the manuscript again to be sure that the English used is clear and correct.

Objective Evaluation: Accept after minor revisions are done.
